# Acute kidney injury in hospitalized children with sickle cell anemia

**DOI:** 10.1186/s12882-022-02731-9

**Published:** 2022-03-18

**Authors:** Anthony Batte, Sahit Menon, John Ssenkusu, Sarah Kiguli, Robert Kalyesubula, Joseph Lubega, Edrisa Ibrahim Mutebi, Robert O. Opoka, Chandy C. John, Michelle C. Starr, Andrea L. Conroy

**Affiliations:** 1 Child Health and Development Centre, Makerere University College of Health Sciences, P.O Box 6717, Kampala, Uganda; 2 San Diego School of Medicine, University of California, San Diego, USA; 3 Department of Epidemiology and Biostatistics, Makerere University School of Public Health, Kampala, Uganda; 4 Department of Paediatrics and Child Health, Makerere University College of Health Sciences, Kampala, Uganda; 5 Makerere University College of Health Sciences, Kampala, Uganda; 6 Pediatric Hematology and Oncology, Baylor College of Medicine, Texas, USA; 7 Department of Pediatrics, Ryan White Center for Pediatric Infectious Disease and Global Health, Indiana University School of Medicine, Indianapolis, IN USA; 8 Department of Pediatrics, Division of Nephrology, Indiana University School of Medicine, Indianapolis, IN USA

**Keywords:** Sickle cell anemia, Acute kidney injury, Children, Sub-Saharan Africa, Vaso-occlusive crises, Malaria, Infection, Hemoglobinuria, Cystatin C, Serum creatinine

## Abstract

**Background:**

Children with sickle cell anemia (SCA) are at increased risk of acute kidney injury (AKI) that may lead to death or chronic kidney disease. This study evaluated AKI prevalence and risk factors in children with SCA hospitalized with a vaso-occlusive crisis (VOC) in a low-resource setting. Further, we evaluated whether modifications to the Kidney Disease: Improving Global Outcomes (KDIGO) definition would influence clinical outcomes of AKI in children with SCA hospitalized with a VOC.

**Methods:**

We prospectively enrolled 185 children from 2 – 18 years of age with SCA (Hemoglobin SS) hospitalized with a VOC at a tertiary hospital in Uganda. Kidney function was assessed on admission, 24–48 h of hospitalization, and day 7 or discharge. Creatinine was measured enzymatically using an isotype-dilution mass spectrometry traceable method. AKI was defined using the original-KDIGO definition as ≥ 1.5-fold change in creatinine within seven days or an absolute change of ≥ 0.3 mg/dl within 48 h. The SCA modified-KDIGO (sKDIGO) definition excluded children with a 1.5-fold change in creatinine from 0.2 mg/dL to 0.3 mg/dL.

**Results:**

Using KDIGO, 90/185 (48.7%) children had AKI with 61/185 (33.0%) AKI cases present on admission, and 29/124 (23.4%) cases of incident AKI. Overall, 23 children with AKI had a 1.5-fold increase in creatinine from 0.2 mg/dL to 0.3 m/dL. Using the sKDIGO-definition, 67/185 (36.2%) children had AKI with 43/185 (23.2%) cases on admission, and 24/142 (16.9%) cases of incident AKI. The sKDIGO definition, but not the original-KDIGO definition, was associated with increased mortality (0.9% vs. 7.5%, *p* = 0.024). Using logistic regression, AKI risk factors included age (aOR, 1.10, 95% CI 1.10, 1.20), hypovolemia (aOR, 2.98, 95% CI 1.08, 8.23), tender hepatomegaly (aOR, 2.46, 95% CI 1.05, 5.81), and infection (aOR, 2.63, 95% CI 1.19, 5.81) (*p* < 0.05).

**Conclusion:**

These results demonstrate that AKI is a common complication in children with SCA admitted with VOC. The sKDIGO definition of AKI in children with SCA was a better predictor of clinical outcomes in children. There is need for promotion of targeted interventions to ensure early identification and treatment of AKI in children with SCA.

## Background

Sickle cell anemia (SCA) is an inherited hemoglobinopathy that disproportionately impacts children in sub-Saharan Africa. Globally, 300,000 children are born with SCA annually with 80% of affected children residing in Africa [1, 2]. Vaso-occlusive pain crises (VOC) are a common complication in children with SCA [3] and a risk factor for AKI with an estimated 2.5–17% of children hospitalized with a VOC developing AKI in high-income countries (HIC) [4–7]. Risk factors for AKI in children with VOC include hypovolemia, and exposure to nephrotoxic medications, including non-steroidal anti-inflammatory drugs for pain management and aminoglycosides for the management of suspected infections [8, 9]. However, there are limited data on AKI prevalence, risk factors and outcomes in children with SCA and VOC in LMIC.

AKI is a sudden change in kidney function denoted by either a change in serum creatinine or urine output. AKI is defined as a 1.5-fold increase in serum creatinine from estimated baseline, a 0.3 mg/dL increase in serum creatinine (SCr) over 48 h, or a decrease in urine output according to the Kidney Disease: Improving Global Outcomes (KDIGO) criteria [10]. Children with SCA have underlying abnormalities in kidney function including hyperfiltration [11], resulting in baseline SCr levels that are lower than expected for age. This makes defining AKI challenging in this population and raises questions about whether a 1.5-fold increase in SCr is clinically relevant in populations with low baseline SCr.

To address these questions, we conducted a prospective observational study of children with SCA hospitalized with a VOC. We hypothesized that modifications to the KDIGO definition in the context of a population of children with SCA and low baseline SCr levels would be associated with more clinically meaningful changes in AKI outcomes. The primary objective of this study was to define AKI prevalence and risk factors in children with SCA hospitalized with a VOC. Our secondary objective was to evaluate differences in AKI biomarkers (Cystatin C) and outcomes in children with the original and sickle cell modified KDIGO definition.

## Materials and methods

### Study design and setting

Between January and August in 2019, children admitted to Mulago National Referral Hospital with SCA and VOC were screened for eligibility. Eligible children were prospectively recruited in a hospital-based study to define AKI prevalence, risk factors, and outcomes. Inclusion criteria were hospital admission, documented SCA by hemoglobin electrophoresis, age 2 to 18 years, a pain score ≥ 2 using an age-specific pain scale, and a willingness to complete study procedures. Pain in children aged 2–3 years was assessed using the face, legs, activity, cry, and consolability (FLACC) scale [12], the Wong-Baker Faces pain scale in children 3–7 years of age, and the numeric pain scale in children ≥ 8 years [13].

Children were recruited from the paediatric emergency unit that admits an average of 3 children daily with SCA and VOC. Most of the children admitted with VOC also attend the hospital's dedicated sickle cell clinic that provides medical care to approximately 1400 children per year. Routine care includes malaria prophylaxis using sulfadoxine-pyrimethamine, daily folic acid, and penicillin V for children < 5 years of age. Only a small proportion of children receive hydroxyurea.

The sample size was calculated using the formula $n=\left({z}^{2}p\left(1-p\right)/{d}^{2}\right)$, where; z = standard normal variate corresponding to the 95% confidence interval and is 1.96, d = the required precision of the estimate (0.05), p = prevalence rate [14]. The sample size was calculated based on previous studies indicating AKI prevalence of 2.3%, 6.9%, and 13% among children with SCA and VOC [6], generating a sample size of 173.

### Study procedures

After informed consent and enrolment, all children had a complete history and physical examination conducted by a study doctor to assess medication use, signs of infection, the site and severity of pain. Blood pressure was calculated as the mean of three independent measurements and hypertension was defined as a systolic blood pressure > 95% percentile and/or a diastolic blood pressure > 95% for children < 13 years of age or a systolic blood pressure ≥ 130 mmHg or diastolic blood pressure ≥ 80 mmHg for children 13 years of age or older [15]. Liver size was evaluated by abdominal palpation below the subcostal margin and defined as: i) no hepatomegaly, liver not palpable; ii) hepatomegaly, liver palpable below the costal margin; iii) tender hepatomegaly, tenderness on palpation of the liver. A spot urine sample was collected from each recruited child for dipstick urinalysis, urine microscopy, assessment of urine albumin and urine creatinine. Microalbuminuria was defined as urine albumin-to-creatinine ratio of 3-30 mg/mmol and macroalbuminuria as urine albumin-to-creatinine ratio, > 30 mg/mmol.

Children were assessed for symptoms, signs of infection and had a complete blood count and malaria evaluation by Giemsa microscopy. Sepsis was defined using International paediatric sepsis consensus guidelines [16] based on two or more of the following criteria, one of which must be abnormal temperature > 38.5 °C or < 36 °C: age-specific tachycardia, age-specific tachypnea, or leukocytosis. We used age-specific thresholds for leukocytosis in children with SCA [17]. We categorised children as having hypovolemia if they were unable to drink or breastfeed, had diarrhea or vomiting, were hypotensive, or capillary refill time > 3 s (features of dehydration). Children were diagnosed with a urinary tract infection based on a positive nitrite or leukocyte test by urinalysis in a child with a history of fever. An acute infection was defined by the presence of sepsis, malaria or urinary tract infection. Microbiology to culture blood or urine samples was not feasible.

### Measurement of Cystatin C

Cystatin C— a biomarker of glomerular filtration [6] — was measured on cryopreserved serum using an ELISA correlated to the reference standard (Catalog # ERM-DA471/IFCC) with a slope of 1.07 and *R*^2^ value of 0.998 (Quantikine® immunoassay, R&D Systems, Minneapolis, MN) [18]. The assay has been evaluated for matrix effects using samples from apparently healthy donors with a mean (SD) of 0.79 mg/L (1.6) for serum and 0.77 mg/L (1.6) for EDTA plasma [18]. On each plate pooled serum samples were tested in duplicate alongside a commercial control (QC23: Quantikine Immunoassay Control Group 8, R&D Systems, Minneapolis, MN). Laboratory technicians were blinded to participant details.

### Assessment of kidney function

AKI was defined based on the Kidney Disease: Improving Global Outcomes (KDIGO) guidelines as an increase in SCr of ≥ 0.3 mg/dl within 48 h or a 50% increase in baseline creatinine within 7 days [10]. Daily 24 h urine output was not quantified over hospitalization. SCr was assessed in whole blood on enrolment, at 24–48 h, and day 7 or discharge (whichever happened earlier) using iSTAT CHEM8 + cartridges (Abbott Point of Care Inc., Princeton, NJ). This approach of SCr measurement uses an enzymatic assay with values traceable to the U.S. National Institute of Standards and Technology (NIST) standard reference material SRM909, with a reportable range of 0.20–20.0 mg/dL. To define AKI, SCr values below the reportable range were assigned a value of 0.19 mg/dL. Each participant’s lowest measured creatinine, or the nadir, was taken as the baseline SCr for AKI determination. None of the patients had a known baseline creatinine prior to hospitalization. As SCA is a risk factor for kidney disease, population-based estimates of baseline serum creatinine would be inaccurate in this patient population. In instances where a child had only a single creatinine measure (*n* = 7), the Pottel height-independent GFR estimating equation was used to back-calculate baseline creatinine, assuming a normal GFR of 120 mL/min per 1.73m^2^ [19, 20].

Using the KDIGO guidelines, AKI was staged based on creatinine fold change from the nadir to the maximum, where: stage 1 included a 1.5- <2.0-fold change in creatinine from baseline or a ≥ 0.3 mg/dl increase in creatinine within 48 h; stage 2, 2– <3.0-fold change in serum creatinine from baseline; stage 3, ≥3.0-fold change in creatinine from baseline, or an increase in creatinine to ≥ 4.0 mg/dl, or an eGFR ≤ 35 ml/min per 1.73 m^2^. AKI was assessed in two ways: using the original KDIGO definition and using a sickle cell anemia modified KDIGO definition (sKDIGO). The point-of-care i-STAT system reports creatinine in 0.1 mg/dL increments so a one-unit change in creatinine from 0.2 to 0.3 mg/L constituted AKI using the KDIGO definition. In the sKDIGO definition, children with a nadir creatinine of 0.2 mg/dL with 1.5-fold increase to 0.3 mg/dL were excluded from the AKI definition. The eGFR was calculated using the CKiD creatinine and Cystatin C based formulae [21]: creatinine, eGFR = (0.413*height)/SCr; Cystatin C, eGFR = 70.69 *(Serum Cystatin C)^−0.931^; SCr & Cystatin C, eGFR = 39.8*((height/SCr)^0.456^)*((1.8/Cystatin C)^0.418^)* ((30/bun) ^0.079^)*(1.076^male^) *((height/1.4)^0.179^).

### Statistical analysis

Data was analysed using STATA v14.0 (StataCorp) and GraphPad Prism v7.03. Data was summarised descriptively using median and interquartile range (IQR) for continuous variables and the number and frequency for discrete variables. Differences between continuous variables and AKI status were assessed using the Wilcoxon rank-sum test or a non-parametric test of trend across stages of AKI. Differences between categorical variables were analysed using the Pearson’s Chi-square test or Fisher’s exact test, as appropriate. To identify risk factors for AKI we used logistic regression and computed unadjusted and adjusted odds ratios. Variables were considered for inclusion in the multivariable models based on an a priori hypothesized relationship or a *p* < 0.20 in bivariate analysis. Age and sex were included in all models.

## Results

### Characteristics of the study population

**Fig. 1:**
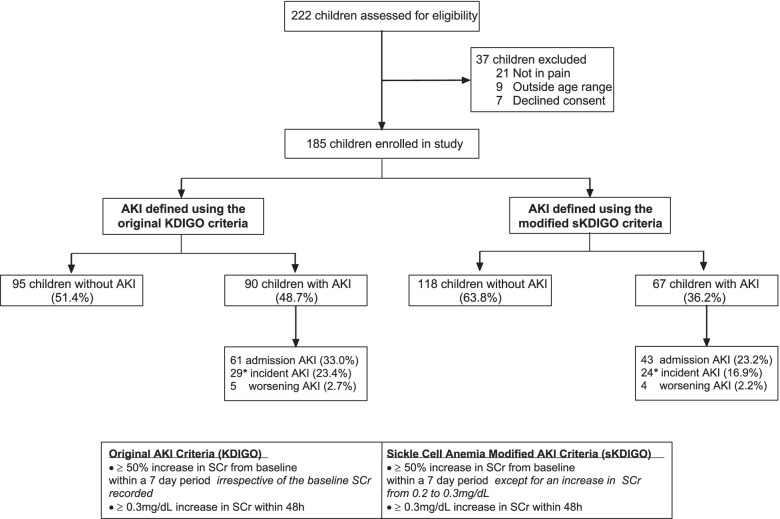
Flow chart of study population. AKI was defined in children according to the original KDIGO definition or a modified definition (sKDIGO) where children with a 1.5-fold increase in serum creatinine (SCr) from 0.2 to 0.3 mg/dL was not considered AKI. AKI status was defined as admission AKI if a child met criteria for AKI using the admission SCr based on the nadir SCr measured over hospitalization or incident AKI in children whom did not have AKI on admission but developed it over hospitalization. When assessing the incidence of AKI over hospitalization, children with AKI present on admission were excluded from the denominator. *Children with a maximum AKI stage higher than the admission AKI stage were considered to have worsening AKI

A total of 185 children with SCA and VOC were enrolled into the study (Fig. 1). The median age was 8.9 years (IQR, 5.9—11.8) and 41.6% of participants were female (Table 1). The prevalence of undernutrition was high with 30.8% of children stunted (height-for-age z score < –2), 25.9% underweight (weight-for-age z score < -2), and 29.7% wasted (weight-for-height z score or BMI-for-age z score < -2). Of the 185 children enrolled, 24 (13.0%) children had a history of stroke, and 78 (42.2%) had been hospitalized in the previous six months (Table 1). The majority (70.3%) of children had severe anemia with a hemoglobin < 8 g/dL and 38 (20.5%) children had evidence of an acute infection defined as sepsis (*n* = 19, 10.3%), malaria (*n* = 17, 9.6%), or urinary tract infection (*n* = 4, 2.2%). Six (3.2%) of the participants died during hospitalization and all deaths occurred in female participants (mortality, 7.8%).

**Table 1:** Demographic and clinical characteristics of the participants

	**N (% missing)**	**Study population (*n* = 185)**
**Demographics**		
Age, years, median (IQR)	185 (0.0)	8.9 (5.9, 11.8)
Age categories, n (%)		
< 5 years	185 (0.0)	36 (19.5)
5–10 years		73 (39.5)
> 10 years		76 (41.1)
Sex, n (%) Female	185 (0.0)	77 (41.6)
Height-for-age z score	184 (0.1)	-1.36 (-2.28, -0.40)
Weight-for-age z score^a^	112 (39.5)	-1.47 (-2.04, -0.47)
Weight-for-height z score^a^	38 (79.5)	-1.59 (-2.19, -0.39)
BMI-for-age z score^a^	149 (19.5)	-1.31(-2.26, -0.40)
HIV infection, n (%)	185 (0.0)	1 (0.5)
**Routine Medication Use**		
Folic acid, n (%)	185 (0.0)	117 (63.2)
Hydroxyurea, n (%)	185 (0.0)	61 (33.0)
Penicillin V prophylaxis, n (%)	185 (0.0)	11 (6.0)
Children < 5 years of age	36 (0.0)	5 (13.9)
**Medical History**		
Hospital admission past 6 months, n (%)	185 (0.0)	78 (42.2)
Prior transfusion, n (%)	185 (0.0)	140 (75.7)
History of stroke, n (%)	185 (0.0)	24 (13)
**Clinical characteristics on admission**		
Fever, n (%)	185 (0.0)	64 (34.6)
Heart rate, bpm	185 (0.0)	108 (98, 121)
Blood pressure category, n (%)		
Hypotensive	185 (0.0)	2 (1.1)
Normotensive		149 (80.5)
Hypertension		31 (18.4)
Diarrhea, n (%)	185 (0.0)	4 (2.2)
Vomiting, n (%)	185 (0.0)	13 (7.0)
Unable to drink/breastfeed, n (%)	185 (0.0)	8 (4.3)
Severe anemia, Hemoglobin < 8.0 g/dL, n (%)	185 (0.0)	130 (70.3)
Splenomegaly, n (%)	179 (3.2)	18 (9.7)
Liver assessment		
No hepatomegaly	185 (0.0)	107 (57.8)
Hepatomegaly		40 (21.6)
Tender Hepatomegaly		38 (20.5)
History of reduced urine output, n (%)	185 (0.0)	15 (8.1)
History of tea coloured urine, n (%)	185 (0.0)	79 (42.7)
**Laboratory findings**		
WBC × 10^3^/μL	185 (0.0)	22.6 (16.7, 33.4)
Neutrophil count × 10^3^/μL	185 (0.0)	12.1 (8.3, 17.2)
Hemoglobin, g/dL	185 (0.0)	7.3 (6.3, 8.3)
Platelet count × 10^9^/L	185 (0.0)	418 (306, 525)
Sepsis, n (%)	185 (0.0)	19 (10.3)
Malaria, n (%)	177 (4.3)	17 (9.6)
Urinary tract infection, n (%)	182 (1.6)	4 (2.2)
Acute infection, n (%)	185 (0.0)	38 (20.5)
**Kidney Function**		
Creatinine, mg/dL	185 (0.0)	0.3 (0.19, 0.4)
Urine albumin to creatinine ratio, n (%)		
< 3 mg/mmol	175 (5.4)	105 (60.0)
3–30 mg/mmol		55 (31.4)
> 30 mg/mmol		15 (8.6)
Admission eGFR, mL/min per 1.73m^2^		
CKiD SCr equation	184 (0.1)	184 (139, 227)
CKiD Cystatin C equation	184 (0.1)	86 (68, 107)
CKiD SCr + Cystatin C equation	184 (0.1)	133 (104, 160)
**Outcome**		
In-hospital mortality, n (%)	185 (0.0)	6 (3.2)

Data presented as median (IQR) or n (%)

^a^ Weight-for-age available for children ≤ 10 years of age (*n* = 113); weight-for-height for children < 5 years or age, BMI-for-age for children ≥ 5 years of age (WHO standards, 2009)

The median duration of pain prior to admission was 3 days. The reported locations of pain included the lower limbs (64.9%), abdomen (41.6%), upper limbs (30.3%), back (29.7%), chest (34.6%) and others (3.8%). Analgesia use for children included paracetamol (93.0%), morphine (86.0%), ibuprofen (83.8%), diclophenac (10.3%), codeine (2.2%) and tramadol (1.1%).

### Prevalence of AKI in children with SCA

**Fig. 2:**
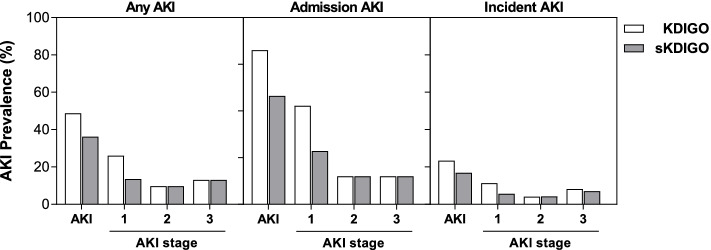
AKI prevalence and stage based on KDIGO or sKDIGO definition. The prevalence of AKI and AKI stage over hospitalization (any AKI), admission, and incident AKI (developed in-hospital). Creatinine was measured on admission, 24–48 h of hospitalization, and discharge or day 7 (whichever happened earlier). For sKDIGO definition children with a 50% increase in creatinine from a baseline measure of 0.2 mg/dL were not considered to have AKI

Overall, 90/185 (48.7%) children had AKI using the KDIGO definition with the majority of AKI cases occurring on admission 61/90 (67.8%) while 34/90 (37.8%) children had incident or worsening AKI over hospitalization (Fig. 2). In children with AKI, 23/90 (25.6%) of cases occurred in children with a baseline creatinine of 0.2 mg/dL that increased to 0.3 mg/dL over hospitalization. Using the sKDIGO definition, 67/185 (36.2%) children had AKI with 26 cases diagnosed based on a 0.3 mg/dL increase in creatinine within 48 h and 41 cases based on a fold increase in creatinine over 7 days; 43/67(64.2%) had AKI on admission and 28/67 (41.8%) developed incident or worsening AKI during hospitalization (Fig. 2). There were no relationships between medication use and AKI (*p* > 0.05 for all).

### Comparison of laboratory findings and clinical outcomes based on KDIGO or sKDIGO definition of AKI

**Fig. 3:**
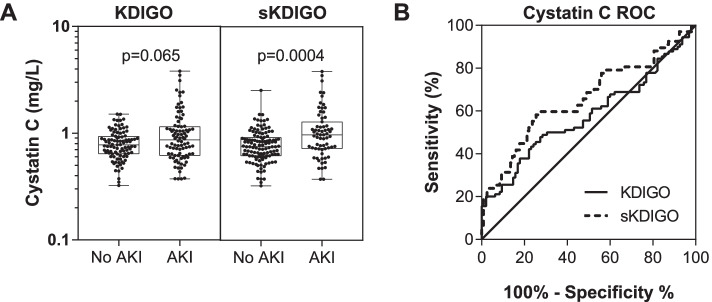
Comparison of Cystatin C levels based on the KDIGO AKI definition. **A** Box and whisker plots showing the median (interquartile range) and range of serum Cystatin C with the individual data points overlaid. Differences between groups were compared using Wilcoxon rank-sum test with the *p* values presented on the graphs. **B** Receiver operating characteristic (ROC) curve to measure the association between the biomarker level and AKI status. Serum Cystatin C had a higher area under the receiver operating characteristic (ROC) curve (AUROC) using sKDIGO compared to KDIGO

We evaluated the relationship between both AKI definitions and clinical chemistries and urinalysis results (Table 2). Overall, differences in laboratory parameters were larger in children who had AKI defined using sKDIGO definition compared to KDIGO (Table 2). We further evaluated the KDIGO and sKDIGO definition using Cystatin C, an alternative marker of GFR (Fig. 3). Cystatin C was only elevated in children meeting the sKDIGO definition of AKI (*p* < 0.001).

**Table 2:** Laboratory findings in children based on acute kidney injury definition

	**KDIGO** ** ≥ 50% from baseline (nadir) or 0.3 mg/dL increase in SCr**			^**a**^**sKDIGO** ** ≥ 50% from baseline (nadir, excluding 0.2 mg/dL to 0.3md/dL) or 0.3 mg/dL increase in SCr**		
	**No AKI (*n* = 95)**	**AKI (*n* = 90)**	***P* value**	**No AKI (*n* = 118)**	**AKI (*n* = 67)**	***P* value**
**Laboratory Findings**						
WBC × 10^3^/μL	22.2 (16.2, 32.4)	24.3 (16.7, 39.2)	0.5257	20.1 (15.4, 30.3)	25.5 (18.2, 46.7)	0.0072
Hemoglobin, g/dL	7.4 (6.4, 8.3)	7.1 (6.1, 8.0)	0.3362	7.4 (6.5, 8.4)	6.9 (5.9, 7.7)	0.0068
Neutrophil count × 10^3^/μL	11.4 (8.2, 16.4)	13.4 (8.4, 19.2)	0.2577	11.3 (8.0, 16.2)	14.3 (8.9, 20.4)	0.0143
Platelet count × 10^3^/μL	439 (334, 560)	387 (282, 505)	0.0361	435 (330, 557)	382 (265, 488)	0.0529
BUN, mg/dL	3 (3, 4)	5 (3, 9)	< 0.0001	3 (3, 4)	6 (3, 11)	< 0.0001
Liver function						
Aspartate transaminase (AST)	43.0 (33.0, 68.0)	65 (39.8, 120.0)	0.0001	43.5 (33.0, 70.0)	67 (48, 148)	< 0.0001
Alkaline phosphatase (ALP)	207 (171, 256)	247 (170, 321)	0.0360	211 (165, 255)	254 (183, 337)	0.0049
Gamma glutamyl transpeptidase (GGT), IU/L	29 (17.2, 49.2)	35.2 (23.6, 74.5)	0.0418	27.7 (16.5, 49.1)	40.4 (25.3, 82.0)	0.0007
Albumin, mg/dL	39.7 (35.8, 43.2)	38.6 (33.7, 42.5)	0.1361	39.7 (35.9, 43.3)	37.8 (32.9, 42.0)	0.0323
Total bilirubin, mg/dL	1.7 (1.0, 2.9)	2.5 (1.1, 4.6)	0.0224	1.6 (0.9, 2.8)	3.1 (1.3, 5.1)	0.0008
Conjugated bilirubin, mg/dL	0.6 (0.5, 1.0)	0.8 (0.5, 2.6)	0.0445	0.6 (0.5, 1.0)	1.1 (0.5, 3.9)	0.0008
Creatinine measures						
Admission SCr, mg/dL	0.2 (0.19, 0.3)	0.3 (0.3, 0.5)	< 0.0001	0.2 (0.19, 0.3)	0.4 (0.3, 0.6)	< 0.0001
Maximum SCr, mg/dL	0.2 (0.19, 0.3)	0.4 (0.3, 1.0)	< 0.0001	0.2 (0.2, 0.3)	0.6 (0.3, 1.5)	< 0.0001
Minimum SCr, mg/dL	0.19 (0.19, 0.3)	0.2 (0.19, 0.3)	0.0087	0.2 (0.19, 0.3)	0.2 (0.19, 0.4)	0.0543
Urinalysis						
uAlbumin:uCr ratio, mg/mmol	1.8 (0.7, 6.5)	2.1 (0.8, 15.1)	0.2706	1.4 (0.7, 3.9)	3.1 (0.9, 23.1)	0.0034
Albumin-to-creatinine ratio, n (%)						
< 3 mg/mmol	54 (60.0)	51 (60.0)	0.607	74 (66.7)	31 (48.4)	0.030
3–30 mg/mmol	30 (33.3)	25 (29.4)		31 (27.9)	24 (37.5)	
> 30 mg/mmol	6 (6.7)	9 (10.6)		6 (5.4)	9 (14.1)	
Hematuria by dipstick, n (%)	4 (4.2)	10 (11.1)	0.076	4 (3.4)	10 (14.9)	0.009
Proteinuria by dipstick, n (%)	8 (8.4)	20 (22.2)	0.009	9 (7.6)	19 (28.4)	< 0.001
Bilirubinuria by dipstick, n (%)	3 (3.2)	13 (14.4)	0.006	3 (2.5)	13 (19.4)	0.001

Continuous data presented as median (IQR) and analyzed using the Wilcoxon rank sum test

Categorical data analyzed using Pearson’s Chi square test or Fisher’s exact test (if *n* < 5 per cell)

^a^Children with a baseline creatinine of 0.2 mg/dL with a 50% increase in creatinine to 0.3 mg/dL were not considered to have AKI

Finally, we compared outcomes in children based on AKI status. AKI was associated with increased mortality using sKDIGO (mortality, 0.9% no AKI vs. 7.5% AKI, *p* = 0.024) but not the original-KDIGO definition (mortality, no AKI, 1.1% vs. 5.6% AKI, *p* = 0.110). AKI on admission was not associated with increased mortality. Rather, children who developed incident AKI in-hospital or those with worsening AKI had increased mortality (*p* < 0.05 for both). As the sKDIGO definitions was a better predictor of mortality, subsequent analyses evaluating risk factors for AKI and AKI progression are conducted using sKDIGO.

### Risk factors for AKI in children with SCA and VOC

In order to improve recognition of AKI in children with SCA who require hospitalization, we evaluated differences in the demographic characteristics, signs and symptoms on admission and laboratory findings in children with AKI (Table 3). As 43/67 (64.2%) children had AKI on admission to hospital, we are unable to attribute causality to these risk factors. However, understanding risk factors in this high-risk population may enable clinicians to identify features that raise their index of suspicion minimizing exposure to potential nephrotoxic agents and prioritizing monitoring in children at highest risk of AKI. Using logistic regression, AKI risk factors included age (aOR, 1.10, 95% CI 1.01, 1.20), hypovolemia (aOR, 2.98, 95% CI 1.08, 8.24), tender hepatomegaly (aOR, 2.46, 95% CI 1.05, 5.77), and an acute infection (aOR, 2.63, 95% CI 1.19, 5.81) (*p* < 0.05).

**Table 3:** Risk factors for AKI in children admitted to hospital for a vaso-occlusive crisis (sKDIGO)

	**Bivariate analysis**				**Multivariate analysis**	
	**No AKI (*n* = 118)**	AKI* (*n* = 67)	**OR (95% CI)**	***P* value**	**aOR (95% CI)**	***P* value**
**Demographics and Clinical Characteristics**						
Age, years, median (IQR)	8.0 95.1, 11.3)	10 (7.3, 12.4)	1.11 (1.03–1.20)	0.010	**1.10 (1.10, 1.20)**	**0.022**
Age categories, n (%)						
< 5 years	28 (23.7)	8 (11.9)	1 (reference)			
5–10 years	47 39.8)	26 (38.8)	1.94 (0.77–4.86)	0.159		
> 10 years	43 (36.4)	33 (49.3)	2.69 (1.08–6.65)	0.033		
Sex, n (%) Female	42 (35.6)	35 (52.2)	1.98 (1.08–3.64)	0.028	1.74 (0.88, 3.45	0.110
Height-for-age z score	-1.2 (-2.1, -0.3)	-1.7 (-2.6, -1.0)	0.8 (0.64–0.99)	0.044		
Blood pressure category, n (%)						
Hypotensive	1 (0.9)	1 (1.5)	1.81 (0.11–29.55)	0.677		
Normotensive (reference)	96 (81.4)	53 (79.1)	1 (reference)	–-		
Hypertensive	21 (17.8)	13 (19.4)	1.12 (0.52–2.42)	0.770		
Diarrhea, n (%)	1 (0.9)	3 (4.5)	5.48 (0.56–53.81)	0.144		
Vomiting, n (%)	5 (4.2)	8 (11.9)	3.06 (0.96–9.78)	0.059		
Unable to drink/breastfeed, n (%)	1 (0.9)	7 (10.5)	13.65 (1.64–113.52)	0.016		
Hypovolemia (%)	8 (6.8)	16 (23.9)	4.31 (1.73–10.73)	0.002	**2.98 (1.08, 8.23)**	**0.035**
Severe anemia, hemoglobin < 8.0 g/dL, n (%)	77 (65.3)	54 (80.6)	2.21 (1.08–4.52)	0.029		
Splenomegaly, n (%)	9 (7.6)	9 (13.4)	1.88 (0.70–4.99)	0.208		
No hepatomegaly	77 (65.3)	30 (44.8)	1 (reference)	–-	1 (reference)	–-
Hepatomegaly	24 (20.3)	16 (23.9)	1.71 (0.80–3.66)	0.166	1.57 (0.69, 3.59)	0.281
Tender hepatomegaly	17 (14.4)	21 (31.3)	3.17 (1.73–6.82)	0.003	**2.46 (1.05, 5.77)**	**0.038**
History of reduced urine output, n (%)	8 (6.8)	7 (10.5)	1.60 (0.55–4.63)	0.383		
History of tea coloured urine, n (%)	41 (34.8)	38 (56.7)	2.46 (1.33–4.55)	0.004	1.63 (0.83, 3.22)	0.157
**Laboratory Findings**						
WBC × 10^3^/μL	20.1 (15.4, 30.3)	25.5 (18.2, 46.7)	1.01 (1.002–1.026)	0.015		
Neutrophil count × 10^3^/μL	11.3 (8.0, 16.2)	14.3 (8.9, 20.4)	1.04 (1.004–1.072)	0.029		
Hemoglobin, g/dL	7.4 (6.5, 8.4)	6.9 (5.9, 7.7)	0.79 (0.65–0.97)	0.022		
Platelet count × 10^3^/μL	435 (330, 557)	382 (265, 488)	1.00 (1.00–1.00)	0.079		
Sepsis, n (%)	9 (7.6)	10 (14.9)	2.12 (0.82–5.52)	0.122		
Malaria, n (%)	7 (6.3)	10 (15.2)	2.65 (0.96–7.35)	0.061		
Urinary tract infection, n (%)	1 (0.9)	3 (4.6)	5.48 (0.56–53.74)	0.145		
Acute infection, n (%)	17 (14.4)	21 (31.3)	2.71 (1.31–5.62)	0.007	**2.63 (1.19, 5.81)**	**0.017**
Urine ACR, n (%)						
< 3 mg/mmol	74 (66.7)	31 (48.4)	1 (reference)	–-		
3–30 mg/mmol	31 (27.9)	24 (37.5)	1.85 (0.94–3.64)	0.076		
> 30 mg/mmol	6 (5.4)	9 (14.1)	3.58 (1.17–10.92)	0.025		

Variables in the multivariate analysis included age, sex, hypovolemia, hepatomegaly, reported history of tea coloured urine, and an acute infection

### Risk factors for AKI on admission and worsening or incident AKI during hospitalization

To determine risk factors in children with community-acquired AKI versus hospital-acquired or worsening AKI, we stratified analyses based on the timing of diagnosis (Table 4). Children with community-acquired AKI were older, were more likely to have tea coloured urine, and hepatomegaly or tender hepatomegaly. In addition, AKI on admission was associated with hypovolemia and an acute infection and laboratory findings consistent with an acute inflammatory event. In contrast, participants with incident or worsening AKI over hospitalization were similar in age, with comparable frequencies of infection and similar laboratory findings. There were no differences in nephrotoxic medications use in children with incident or worsening AKI (Table 4). Participants who developed incident or worsening AKI were more likely to be female.

**Table 4:** Risk factors of AKI on admission and incident or worsening AKI using sKDIGO

		**Admission AKI**			**Incident or worsening AKI**		
		**No AKI (*n* = 142)**	**AKI (*n* = 43)**	***P* value**	**No AKI (*n* = 118)**	**AKI (*n* = 28)**	***P* value**
**Demographics and Clinical Characteristics**							
Age, years, median (IQR)		**8.3 (5.1, 11.4)**	**10.3 (7.3, 12.3)**	**0.021**	8.0 (5.1, 11.3)	9.6 (5.6, 13.0)	0.264
Sex, n (%) Female		57 (40.1)	20 (46.5)	0.458	**42 (35.6)**	**17 (60.7)**	**0.015**
Blood pressure category, n (%)							
Hypotensive		1 (0.7)	1 (2.3)	0.441	1 (0.9)	0 (0.0)	1.000
Normotensive (reference)		116 (81.7)	33 (76.7)		96 (81.4)	23 (82.1)	
Hypertensive		25 (17.6)	9 (20.9)		21 (17.8)	5 (17.9)	
Diarrhea, n (%)		2 (1.4)	2 (4.7)	0.231	1 (0.9)	1 (3.6)	0.348
Vomiting, n (%)		8 (5.6)	5 (11.6)	0.178	5 (4.2)	3 (10.7)	0.180
Unable to drink/breastfeed, n (%)		**2 (1.4)**	**6 (14.0)**	**0.002**	1 (0.9)	1 (3.6)	0.348
Hypovolemia, n (%)		**13 (9.2)**	**11 (25.6)**	**0.005**	8 (6.8)	5 (17.9)	0.064
Splenomegaly, n (%)		13 (9.2)	5 (11.6)	0.733	9 (7.6)	5 (17.9)	0.252
No hepatomegaly		**92 (64.8)**	**15 (34.9)**	**0.001**	77 (65.3)	16 (57.1)	0.620
Hepatomegaly		**28 (19.7)**	**12 (27.9)**		24 (20.3)	6 (21.4)	
Tender Hepatomegaly		**22 (15.5)**	**16 (37.2)**		17 (14.4)	6 (21.4)	
History of reduce urine output, n (%)		9 (6.3)	6 (14.0)	0.109	8 (6.8)	1 (3.6)	1.000
History of tea coloured urine, n (%)		**53 (37.3)**	**26 (60.5)**	**0.007**	41 (34.8)	14 (50.0)	0.134
**Nephrotoxic medication use in hospital**							
Ibuprofen, n (%)	120 (84.5)		35 (81.4)	0.628	99 (83.9)	24 (85.7)	0.813
Diclophenac, n (%)	16 (11.3)		3 (7.0)	0.571	15 (12.7)	2 (7.1)	0.528
Gentamicin, n (%)	0 (0.0)		1 (2.3)	0.232	0 (0.0)	0 (0.0)	–-
**Laboratory Findings**							
WBC × 10^3^/μL	**21.0 (15.5, 30.2)**		**30.8 (19.3, 51.8)**	**0.0002**	20.1 (15.4, 30.3)	21.7 (15.5, 30.2)	0.980
Neutrophil count × 10^3^/μL	**11.4 (8.0, 16.2)**		**16.1 (10.5, 25.3)**	**0.001**	11.3 (8.0, 16.2)	12.2 (8.0, 16.9)	0.585
Hemoglobin, g/dL	**7.4 (6.5, 8.4)**		**6.2 (5.6, 7.4)**	** < 0.0001**	7.4 (6.5, 8.4)	7.5 (7.0, 8.6)	0.388
Platelet count × 10^3^/μL	**434 (330. 557)**		**372 (251, 449)**	**0.0140**	435 (330, 557)	422 (302, 570)	0.831
Sepsis^5^, n (%)	12 (8.5)		7 (16.3)	0.138	9 (7.6)	3 (10.7)	0.701
Malaria, n (%)	**9 (6.7)**		**8 (19.1)**	**0.017**	7 (6.3)	3 (10.7)	0.421
Urinary tract infection, n (%)	**1 (0.7)**		**3 (7.1)**	**0.039**	1 (0.9)	0	1.000
Acute infection, n (%)	**22 (15.5)**		**16 (37.2)**	**0.002**	17 (14.4)	6 (21.4)	0.359
Hematuria, n (%)	**5 (3.5)**		**9 (20.9)**	** < 0.001**	4 (3.4)	2 (7.1)	0.324
Proteinuria, n (%)	**14 (9.9)**		**14 (32.6)**	**< 0.001**	9 (7.6)	5 (17.9)	0.098
Bilirubinuria, n (%)	**9 (6.3)**		**7 (16.3)**	**0.042**	**3 (2.5)**	**6 (21.4)**	**0.002**
**Pain characteristics**							
Pain Score, median (IQR)	6 (4,8)		6 (4, 8)	0.242	6 (4,8)	6 (4,8)	0.755
Duration of pain in days	3 (2,4)		3 (2,6)	0.126	3 (2,4)	3 (2, 5)	0.978

## Discussion

While AKI is a recognised clinical complication of VOC in children with SCA [8], there is a paucity of prospective studies evaluating the incidence of AKI in children with SCA from LMIC. In this study we used the KDIGO consensus guidelines to define AKI in children with SCA presenting with VOC, using serial creatinine measures over hospitalization. Given the percentage of children that had low creatinine values as a result of hyperfiltration and undernutrition, we also considered sKDIGO definition. Overall, the sKDIGO was associated with higher mortality compared to the original KDIGO definition. Risk factors of AKI on admission included older age, an acute infection, tender hepatomegaly, hypovolemia and tea coloured urine.

SCA is associated with a number of kidney abnormalities including hyperfiltration, papillary necrosis, glomerulosclerosis and chronic kidney disease, which collectively may predispose people living with SCA to AKI [8]. Using sKDIGO, 36.2% of participants had AKI with 13.5% stage 1, 9.7% stage 2 and 13.0% stage 3. The prevalence of AKI in the present study is higher than previous studies that reported an AKI prevalence between 2.5% to 17% in children hospitalised with VOC [4–7]. However, these studies were largely conducted in high income countries. Children with SCA in LMICs may have additional risk factors that affect a child’s risk of developing AKI during hospitalization [9], including endemic infections like malaria that contribute to substantial AKI in Ugandan children [22–24]. The majority of AKI in this study was community-acquired AKI and consistent with global trends of increased community-acquired AKI in LMIC [25].

AKI on admission was associated with being unable to drink or breastfeed, vomiting and diarrhea, which are risk factors for hypovolemia and pre-renal AKI. Infections may increase AKI risk by exacerbating dehydration and hypovolemia through increased insensible losses secondary to fever. Infections— especially malaria and sepsis— are common in children with SCA in LMICs and contribute significant morbidity and mortality in these children [9]. Malaria and sepsis are well-established risk factors for AKI [23, 26] that can exacerbate microvascular dysfunction in VOCs as endothelial activation can lead to microvascular dysfunction, inflammation, and the release of endogenous nephrotoxins like cell-free hemoglobin and free heme that increase oxidative stress and contribute to tubular injury [27–31]. Infections are a risk factor for VOC in children with SCA thus enhanced infection prevention may reduce the burden of SCA-related complications including AKI. Further, as children with SCA receive routine clinical follow-up through the sickle cell clinic, education on preventing dehydration and counselling on how to adequately manage pain and fever at home while minimizing exposure to nephrotoxic agents like non-steroidal anti-inflammatory drugs may reduce the burden of community-acquired AKI.

In the present study all study deaths occurred in females with a history of passing tea coloured urine (a feature of hemoglobinuria), and being female was associated with the development of incident or worsening AKI. Additional studies are needed to understand social and biological determinants of health in this population. There was a trend of increased hemoglobinuria in females (49.4%) compared to males (35.9%). In addition to being a risk factor for mortality, hemoglobinuria was also a risk factor for AKI on admission. Hemoglobinuria is a common complication in children with SCA associated with ongoing hemolysis and is associated with progression to chronic kidney disease (CKD) [32]. Although the etiology of hemoglobinuria in children presenting with AKI maybe attributed to ongoing hemolysis, there are reports of a rising incidence of hemoglobinuria (blackwater fever) in Uganda [33, 34]. Blackwater fever often occurs in children with anemia and a history of malaria infection and is a known risk factor for AKI in severe malaria [35–37] and is associated with increased post-discharge morbidity and mortality [34].

AKI is defined by reduced urine output or an increase in serum creatinine. In the context of this study AKI was defined based on serum creatinine alone, as data on urine output was not available. It is challenging to define AKI in children with SCA as glomerular hyperfiltration and increased tubular secretion of creatinine affect serum creatinine levels [8]. Further, creatinine is affected by non-renal factors like muscle wasting [38–40]. Undernutrition was common in the study with 29.7% of the population wasted. As the pre-morbid baseline creatinine was not known in the study population, we employed the participants’ individual trajectory. Increased access to creatinine testing and more routine creatinine testing in children with SCA would enable more precise classification of AKI in children during acute infections or VOC. In this study Cystatin C was elevated in children with AKI highlighting that AKI is associated with both functional changes in filtration as well as tubular injury [41]. Additional research is needed to evaluate alternative biomarkers of AKI in children with SCA.

This study evaluated AKI during a single hospital admission for a VOC. However, children often have multiple occurrences of VOC annually [42, 43]. Currently, the Uganda clinical guidelines recommend initiation of hydroxyurea in children with frequent VOC (> 5 episodes per year). In the NOHARM trial, 308 VOC were recorded in 88 participants over one year [17]. With a prevalence rate of AKI of 36.2% in children evaluated for a single episode of VOC, children with SCA are at risk of repeated AKI episodes. In a large hospital-based study of Black Americans, multiple pain crises annually were associated with a faster decline in glomerular filtration rates (GFR) compared to patients with SCA without frequent pain crises [44]. AKI is an established risk factor for development of CKD [45–48]. Further, individuals living with CKD are at increased risk of AKI, which may further accelerate progression of the CKD [49]. This study highlights the need for further studies to evaluate the incidence of AKI over several years in children with SCA to understand how AKI affects the development of CKD in LMIC where the majority of children with SCA reside. This information will be important to guide the development of guidelines on clinical follow-up of children at risk of CKD following AKI.

Albuminuria is a risk factor for CKD [50–52] and 20% of study participants had an albumin-to-creatinine ratio > 30 mg/mmol suggestive of glomerular damage [49]. SCA is associated with an early age of CKD onset and a faster decline in GFR [44, 53]. In the present study, 54.6% of children < 5 years of age had albuminuria with 45.5% microalbuminuria and 9.1% macroalbuminuria. This is higher than in an ongoing study following 2582 participants > 3 years of age with SCA across four West African countries, where the prevalence of micro- and macroalbuminuria was 29% and 5% respectively (median age of 14 years) [54]. Additional studies are needed to assess the prevalence of albuminuria in children with SCA in steady state to determine the appropriate age to start screening for albuminuria. There is need for early identification of CKD, appropriate clinical management, and earlier referral to specialist kidney services that could have a measurable improvement on child health and quality of life.

One of the limitations of the study was a lack of steady state SCr measurement to estimate baseline serum creatinine which reflects limitations in routine SCr measurements in the setting. We assessed SCr at pre-specified time points to coincide with the KDIGO definition of AKI and did not have daily SCr measurements or measurements beyond 7 days, which may have resulted in missed AKI episodes over hospitalization. Further, a recent prospective multi-national study (AWARE) assessing AKI in critically ill children in 32 pediatric intensive care units across five continents found AKI defined using SCr alone, failed to identify AKI in 67% of children with low urine output [55]. In this study AKI was defined on the basis of serum creatinine alone and thus AKI may be underestimated as we did not assess urine output. Further studies are needed with broader definitions of AKI such as additional use of urine output to define AKI as well as longer follow up periods to evaluate incident AKI over hospitalization, persistence of the kidney injury and kidney recovery. Recognition and validation of AKI risk factors may facilitate identification of children likely to benefit from creatinine testing and targeted interventions to promote kidney recovery.

This study has several strengths including its prospective design and the serial evaluation of creatinine over hospitalization. By using a participant’s individual trajectory of SCr over hospitalization to define AKI, we avoid misclassifying children with pre-existing chronic kidney disease as having AKI based on population estimates of baseline SCr. There are few prospective studies on AKI in children with SCA in LMIC and this study adds to the existing literature on the complications of SCA in children. In addition, this study included a number of clinical laboratory evaluations and assessed biomarkers of AKI which allowed us to evaluate the utility of different definitions to define AKI. Our study highlights the need for more targeted programs aimed at prevention and management of both community-acquired and hospital-acquired AKI in LMICs.

## Conclusion

Our study demonstrates that AKI is a frequent complication of hospitalized children with SCA and highlights the need for a heightened index of suspicion in children admitted with a VOC irrespective of pain severity. Modified KDIGO guidelines (sKDIGO) that excluded an increase from 0.2 to 0.3 as a 1.5-fold increase in SCr correlated better with biomarkers of acute kidney injury and mortality than standard KDIGO AKI guidelines, highlighting the importance of accounting for the hyperfiltration that occurs in children with SCA when defining AKI. The study builds on existing literature on complications of SCA in LMICs and demonstrates that a significant proportion of the AKI is community-acquired. There is thus need for interventions aimed at prevention and management of AKI in children with SCA especially during VOC which are often recurrent. Simple steps including prompt rehydration in children unable to tolerate oral fluids or with fluid loss as a result of diarrhea or vomiting may reduce AKI progression, and in this high risk population, nephrotoxic medications for pain management should be used judiciously.

## Data Availability

The datasets used and/or analysed during the current study are available from the corresponding author on reasonable request.

## Abbreviations

AKI: Acute kidney injury
AOR: Adjusted odds ratio
CI: Confidence interval
eGFR: Estimated glomerular filtration rate
FLACC: Face, legs, activity, cry, and consolability
IDMS: Isotope dilution mass spectrometry
IQR: Interquartile range
KDIGO: Kidney Disease: Improving Global Outcomes
SCA: Sickle cell anemia
SCr: Serum creatinine
LMIC: Low and middle income countries
NSAIDS: Non-steroidal anti-inflammatory drugs
UTI: Urinary tract infection
VOC: Vaso-occlusive crises
